# Body temperature and mouse scoring systems as surrogate markers of death in cecal ligation and puncture sepsis

**DOI:** 10.1186/s40635-018-0184-3

**Published:** 2018-07-27

**Authors:** Safiah H. C. Mai, Neha Sharma, Andrew C. Kwong, Dhruva J. Dwivedi, Momina Khan, Peter M. Grin, Alison E. Fox-Robichaud, Patricia C. Liaw

**Affiliations:** 1 Thrombosis and Atherosclerosis Research Institute (TaARI), McMaster University, 237 Barton St. E., DBRI Room C5-107, Hamilton, ON L8L 2X2 Canada; 2 Department of Medicine, McMaster University, 1280 Main St. W, Hamilton, ON L8S 4K1 Canada

**Keywords:** Mouse model, Surrogate endpoints, Murine sepsis score, Mouse grimace scale, Mouse clinical assessment score for sepsis, Animal ethics, Humane research, 3Rs, ARRIVE guidelines

## Abstract

**Background:**

Despite increasing ethical standards for conducting animal research, death is still often used as an endpoint in mouse sepsis studies. Recently, the Murine Sepsis Score (MSS), Mouse Clinical Assessment Score for Sepsis (M-CASS), and Mouse Grimace Scale (MGS) were developed as surrogate endpoint scoring systems for assessing pain and disease severity in mice. The objective of our study was to compare the effectiveness of these scoring systems and monitoring of body temperature for predicting disease progression and death in the cecal ligation and puncture (CLP) sepsis model, in order to better inform selection of surrogate endpoints for death in experimental sepsis.

**Methods:**

C57Bl/6J mice were subjected to control sham surgery, or moderate or severe CLP sepsis. All mice were monitored every 4 h for surrogate markers of death using modified versions of the MSS, M-CASS, and MGS scoring systems until 24 h post-operatively, or until endpoint (inability to ambulate) and consequent euthanasia.

**Results:**

Thirty percent of mice subjected to moderate severity CLP reached endpoint by 24 h post-CLP, whereas 100% undergoing severe CLP reached endpoint within 20 h. Modified MSS, M-CASS, and MGS scores all increased, while body temperature decreased, in a time-dependent and sepsis severity-dependent manner, although modified M-CASS scores showed substantial variability. Receiver operating characteristic curves demonstrate that the last recorded body temperature (AUC = 0.88; 95% CI 0.77–0.99), change in body temperature (AUC = 0.89; 95% CI 0.78–0.99), modified M-CASS (AUC = 0.93; 95% CI 0.85–1.00), and modified MSS (AUC = 0.95; 95% CI 0.88–1.01) scores are all robust for predicting death in CLP sepsis, whereas modified MGS (AUC = 0.78; 95% CI 0.63–0.92) is less robust.

**Conclusions:**

The modified MSS and body temperature are effective markers for assessing disease severity and predicting death in the CLP model, and should thus be considered as valid surrogate markers to replace death as an endpoint in mouse CLP sepsis studies.

## Background

Experimental animal models have been created and refined for over 80 years to investigate the development, management, and treatment of sepsis [1], and have contributed to many important advances in our understanding of sepsis pathophysiology [2]. Recognizing the ethical implications of using animals in research, the “3Rs”—Replacement, Refinement, and Reduction—form the guiding principles for ethical standards when conducting animal research [3]. These guidelines exclude death as an endpoint, and suggest the use of surrogate markers of death to establish humane endpoints where possible. Surrogate markers of death involve using criteria related to pain, suffering, and/or illness, such as clinically relevant scoring systems that semi-quantitatively assess the physical appearance and/or behavior of an animal [4–6], in order to gauge the time at which animals should be humanely euthanized [7]. These decisions must be carefully balanced with minimizing premature termination of animal studies, which may lead to incomplete observations and may necessitate use of even more animals [7, 8]. Improvements are necessary to achieve more ethical and humane treatment of animals in research related to critical care medicine [7, 9, 10], and specifically for experimental sepsis research [10], where clear endpoint markers have not been established for the gold standard sepsis model of cecal ligation and puncture (CLP).

Treatment of septic patients relies heavily on the monitoring of vital signs and patients at higher risk of death can be identified through early warning scores, which document changes in body temperature, respiratory rate, heart rate, blood pressure, and level of consciousness of the patient [11–13]. A recent pilot study by our group also suggests that applying the Hamilton Early Warning Score at triage in the emergency department may facilitate earlier identification of patients with sepsis [14]. In experimental sepsis, researchers have designed scoring systems similar to the clinical Early Warning Scores as tools to ethically assess the progression of sepsis in mice: the Murine Sepsis Score (MSS) was developed by Shrum et al. in 2014 using the intraperitoneal fecal slurry injection model of septic shock [4]; the Mouse Clinical Assessment Score for Sepsis (M-CASS) was developed in a pneumonia model of septic shock by Huet et al. in 2013 [5]; and the Mouse Grimace Scale (MGS) was developed by Langford et al. in 2010 for the purposes of assessing pain [6], including post-surgical pain [15], which may be applicable to the CLP model due to its surgical nature of sepsis induction.

Despite the development of these validated scoring systems, there is a paucity of animal sepsis research utilizing these tools. Furthermore, these scoring systems have not been previously compared in the widely utilized and clinically relevant CLP sepsis model, and in contrast to clinical scoring systems [13, 14], none of these experimental scoring systems evaluate body temperature in mice. Therefore, the objective of our study was to compare the MSS, M-CASS, and MGS scoring systems, and investigate the utility of body temperature monitoring, to determine which of these markers are most informative for predicting death in the mouse CLP model of sepsis.

## Methods

### Experimental sepsis: cecal ligation and puncture model

C57Bl/6J mice (*Helicobacter hepaticus*-free) were purchased from Charles River Laboratories (Sherbrooke, Quebec, Canada) and bred at the Thrombosis and Atherosclerosis Research Institute at McMaster University (Hamilton, ON, Canada). Mice were housed in a Helicobacter/Murine Norovirus-negative clean room in individually ventilated cages (Tecniplast Sealsafe Plus system) under 12 h dark/light cycles. Air was filtered through HEPA filters using Touch Slimline air handling units, which guarantee 75 air changes per hour in each cage. The mice were provided with enrichment, and sterilized water and food (Harlan Teklad Rodent Diet #2018) ad libitum. Mice received humane care in accordance with Canadian Council on Animal Care (CCAC) guidelines and all studies were approved by the Animal Research Ethics Board at McMaster University (Hamilton, ON, Canada).

Healthy male C57Bl/6J mice, 8–12 weeks of age and weighing 20–25 g, were randomized to either CLP surgery to induce sepsis (*n* = 23 for moderate severity CLP, *n* = 17 for severe CLP; *n* = 3–4 per group per experiment) or sham surgery as a non-septic control (*n* = 18 total, 3 per experiment). These sample sizes were calculated to detect a 30% or greater difference in survival between CLP groups with 80% power and α = 0.05. Since the majority of CLP studies are conducted using male mice, female mice were not used to reduce the total number of mice needed in this study and to control for the potential effects of estrogen on modulating sepsis severity [16]. In accordance with Rittirsch et al. (Nature Protocols, [17]), we used the C57Bl/6 mouse strain which is most commonly used in CLP sepsis because most genetically manipulated (knockout or transgenic) mice are on this genetic background. Methods for randomization and experimenter blinding were used to reduce allocation, selection, and experimenter biases according to Animal Research: Reporting of In Vivo Experiments (ARRIVE) guidelines [18]. The CLP model used in these studies was adapted from protocols by others [17, 19, 20], and has been extensively utilized by our group [21–23]. Briefly, under isofluorane anesthesia, the abdominal area of the mouse was shaved and sterilized with iodine and 70% ethanol. All mice underwent laparotomy prior to exteriorization of the cecum onto the sterilized abdominal surface. In CLP mice, 1 cm of the cecum was ligated and punctured through-and-through using a sterile 18-gauge needle. For the moderate severity of CLP, 0.5 cm of fecal matter was extruded from each puncture hole to ensure patency, whereas 1 cm of fecal matter was extruded from each puncture hole for the severe CLP model, after which the cecum was returned and both layers of the incision were closed with suture. In sham-operated mice, the cecum was exteriorized and returned to the peritoneal cavity without ligation or puncture. Buprenorphine (0.1 mg/kg, Temgesic) and Ringer’s lactate (1 mL) were administered subcutaneously pre-operatively and every 4 h post-operatively for pain relief and fluid resuscitation, respectively. Following surgery, mice were kept 3/cage together with mice of the same treatment group. Since previous reports show that correction of hypothermia post-CLP surgery affects mortality [24], external heat was provided for all mice through heating blankets placed below half of each cage to allow mice to regulate their own body temperature. Mice were monitored every 4 h until 24 h post-surgery, or until reaching endpoint as characterized by complete inability to ambulate (since death is not acceptable as an endpoint in accordance with CCAC ethics standards). Mice were placed under brief isofluorane anesthesia for body temperature measurements, carried out using a rectal probe thermometer (Harvard Apparatus Homeothermic Monitor, Harvard Apparatus Canada, Saint-Laurent, QC) with a consistent insertion depth of 2 cm.

### Modified Murine Sepsis Score

The Murine Sepsis Score (MSS) system involves observing seven components: appearance, level of consciousness, activity, response to stimulus, eyes, respiratory rate, and respiratory quality [4]. The established MSS score is the average of these seven components. Changes in respiratory rate were not quantifiable by visual inspection in the CLP model over a 24-h study period, and were therefore excluded from the modified MSS (Table 1).

**Table 1:** Modified MSS scoring system for monitoring of surrogate endpoints and assessment of disease severity in mouse CLP sepsis, adapted from Shrum et al. [4]

Murine Sepsis Score (MSS)				
Score	0	1	2	3
Appearance	Smooth coat	Slightly ruffled fur	Majority of fur on back is ruffled	Piloerection, puffy appearance
Level of consciousness	Active	Active, avoids standing upright	Active only when provoked	Non-responsive, even when provoked
Activity	Normal	Suppressed eating, drinking, or running	Stationary	Stationary, even when provoked
Response to stimulus	Normal	Slowed response to auditory or touch stimuli	No response to auditory, slowed response to touch	No response to touch stimuli
Eyes	Open	Not fully open, potentially secretions	Half closed, potential secretions	Mostly or completely closed
Respiration quality	Normal	Periods of labored breathing	Consistently labored breathing	Labored breathing with gasps

### Modified Mouse Clinical Assessment Score for Sepsis

The Mouse Clinical Assessment Score for Sepsis (M-CASS) system involves observation of eight markers: fur aspect, activity, posture, behavior, chest movements, chest sounds, eyelids, and weight loss [5]. The established M-CASS score is an average of these eight components. Weight loss was excluded in the modified M-CASS system in our sepsis studies as changes in body weight are not observable over a 24-h study period. Since the M-CASS score was developed in a pneumonia model where chest sounds are informative for disease progression [5], and chest sounds were not audible in our CLP studies, this component was also excluded from the modified M-CASS (Table 2).

**Table 2:** Modified M-CASS scoring system for monitoring of surrogate endpoints and assessment of disease severity in mouse CLP sepsis, adapted from Huet et al. [5]

Mouse Clinical Assessment Score for Sepsis (M-CASS)				
Score	0	1	2	3
Fur aspect	Normal coat	Slightly ruffled fur	Ruffled fur	Ruffled fur and piloerection
Activity	Normal	Reduced	Only when provoked	Little or none with provocation
Posture	Normal	Hunched, moving freely	Hunched, strained or stiff movement	Hunched, little or no movement
Behavior	Normal	Slow	Abnormal when disturbed or provoked	Abnormal, no relocation
Chest movements	Normal	Mild dyspnea	Moderate dyspnea	Severe dyspnea
Eyelids	Normal, open	Opened when disturbed	Partially closed, even when disturbed	Mostly or completely closed, even when provoked

### Modified Mouse Grimace Scale

The Mouse Grimace Scale (MGS) scoring system involves the scoring of five components: orbital tightening, nose bulge, cheek bulge, ear positioning, and whisker change [6, 15]. The established MGS score is the average of these five components. In contrast to the CD-1 mouse strain (which has white cheeks with pink nose) used to develop the MGS [6], our study used the C57Bl/6J mouse strain (which has black cheeks and black nose) as is standard for the CLP model [17]; nose bulge and cheek bulge were indistinguishable from one another in these mice due to lack of color contrast, and were thus grouped as a single component score (Table 3).

**Table 3:** Modified MGS scoring system for monitoring of surrogate endpoints and assessment of disease severity in mouse CLP sepsis, adapted from Langford et al. [6]

Mouse Grimace Scale (MGS)				
Score	0	1	2	3
Orbital tightening	Eyes open	Eyes slightly closed	Eyes half closed	Eyes closed
Nose and cheek bulge	Normal, flat	Slightly rounded extension of skin around nose bridge	Wrinkled nose or cheeks, slight bulge in cheeks	Obvious, rigid appearing nose and cheek bulge
Ear positioning	Ears flat, back against body	Ears alert, slightly angled from back	Ears partially positioned forward or apart	Ears completely erect, far apart
Whisker change	Normal	Some whiskers erect	Whiskers mostly erect or clumping	All whiskers standing on end

### Scoring

For the purpose of our comparisons, the modified MSS, M-CASS, and MGS component scores were standardized to a four-point scale ranging from 0 to 3 to make relevant comparisons between these modified scoring systems (Tables 1, 2, and 3). Scoring was performed independently by two blinded observers, and the mean of these scores was recorded at each time-point for each component in each animal.

### Statistical analyses

Data are represented as mean ± standard error of the mean (SEM) and were statistically analyzed using Student’s unpaired *t* test, two-way analysis of variance (ANOVA) with Bonferroni post-hoc tests, or Mantel-Cox log-rank test for survival curve analyses. Receiver operating characteristic (ROC) curves were computed using the last documented value for each parameter, or change from baseline to final value for temperature, to compare the predictive value for mice that survived the duration of the study vs. those that were sacrificed due to reaching endpoint. All analyses were performed using GraphPad Prism 5.0 software (La Jolla, CA, USA), and differences between groups were considered significant at *p* < 0.05.

## Results

### Body temperature can assess disease progression and predict death in CLP sepsis

**Fig. 1:**
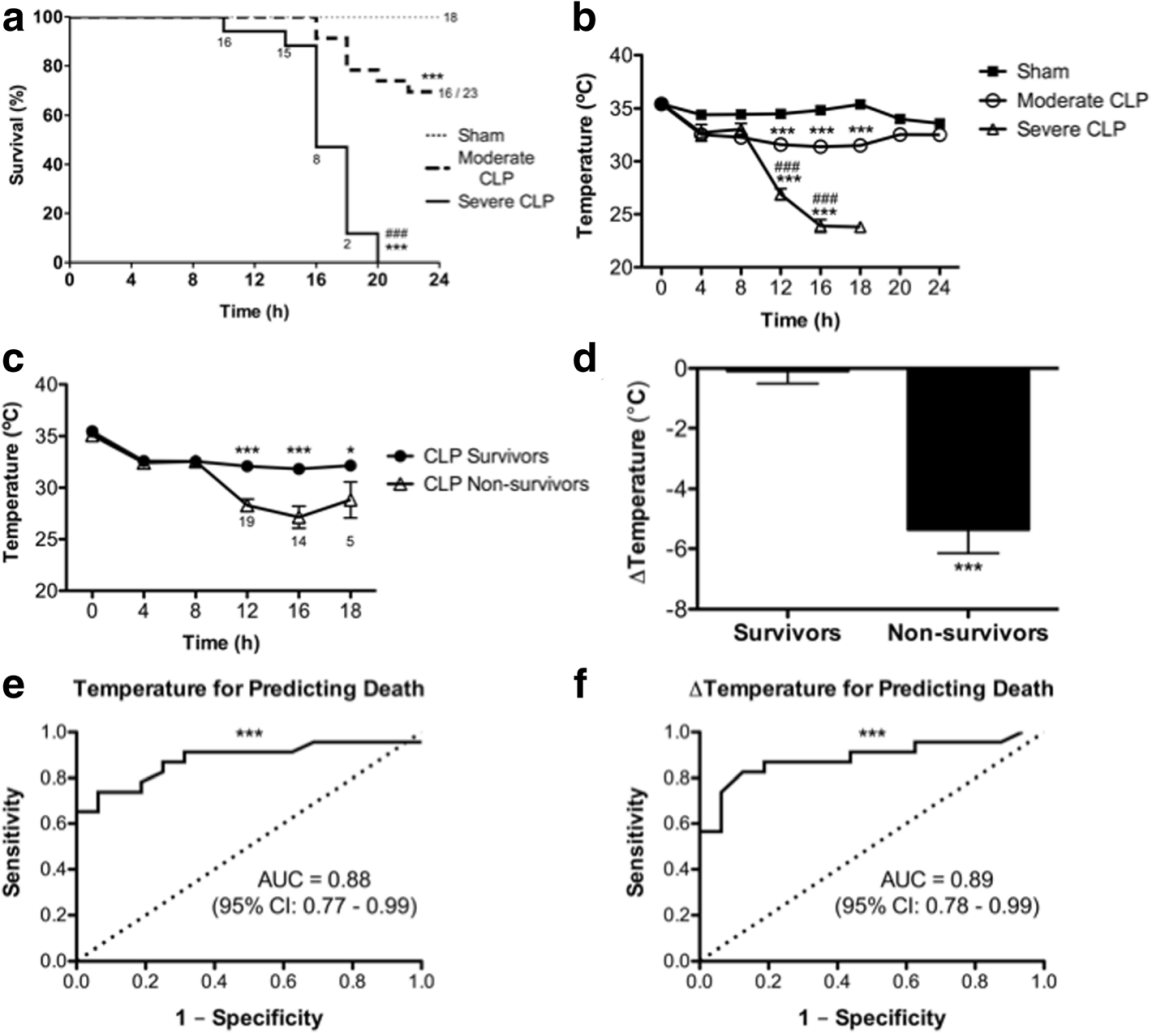
Kaplan-Meier survival curves (**a**) and body temperature over time in sham, moderate, or severe CLP mice (**b**), temperature (**c**), and change in temperature (**d**) over time in survivors vs. non-survivors, and ROC analyses of last body temperature (**e**) and change in body temperature (**f**) as predictors of death in septic mice. Data are presented as mean ± SEM from six independent experiments representing a total number of *n* = 18 sham, *n* = 23 moderate CLP, and *n* = 17 severe CLP mice; *p < 0.05, **p < 0.01, ***p < 0.001 (vs. sham in (**a**, **b**)), ^#^*p* < 0.05, ^##^*p* < 0.01, ^###^*p* < 0.001 vs. moderate CLP, by two-way ANOVA (or by Mantel-Cox log-rank test in (**a**) or *t* test in (**d**)). Note: Only two mice undergoing severe CLP survived past 18 h, therefore statistical analyses were not performed at this time-point in (**b**); similarly in (**c**), time-points past 18 h lacked sufficient *n* in the CLP non-survivors group to perform comparisons, and therefore are not included. Dotted line in (**e**, **f**) represents the line of identity for statistical comparison in ROC analyses. *AUC* area under the curve, *95*% *CI* 95% confidence interval

To determine whether body temperature could be utilized to monitor disease progression in sepsis, mice were subjected to sham surgery, moderate CLP or severe CLP procedures, and body temperature was monitored through a rectal probe every 4 h. During the 24-h study period, severe CLP resulted in 100% mortality by 20 h, which differed significantly from the 30% mortality observed at 24 h in moderate CLP and 0% in sham groups (Fig. 1a). Furthermore, mice subjected to severe CLP had significantly lower body temperature compared to mice undergoing moderate CLP (Fig. 1b), which demonstrates that body temperature can be used as a marker to monitor disease progression in CLP sepsis. Interestingly, body temperature decreased in trends that were parallel to survival, and preceded sharp decreases in survival by approximately 4 h for both CLP groups (compare Fig. 1a, b). These observations led us to compare body temperature over time in CLP mice that survived the full study duration and in non-survivors, the latter exhibiting significantly lower body temperature (< 30 °C) at 12, 16, and 18 h post-CLP (Fig. 1c) and significantly greater changes in body temperature (Fig. 1d). To investigate whether temperature could be used as a predictor of death in the CLP model of sepsis, we conducted ROC analyses using the last recorded body temperature, and using change from baseline to final temperature of survivors and non-survivors. The area under the curve (AUC) of 0.88 (95% confidence interval 0.77–0.99) for last recorded temperature indicates that body temperature < 30 °C (Fig. 1e) is a robust marker for animals that reached endpoint in our study, as is reduction in body temperature (> 5 °C) over time (AUC 0.89, 95% confidence interval 0.78–0.99) in each animal (Fig. 1f). Therefore, body temperature can be used as a surrogate marker for death in CLP sepsis studies.

### Modified Mouse Grimace Scale can assess disease progression, but cannot predict death, in CLP sepsis

**Fig. 2:**
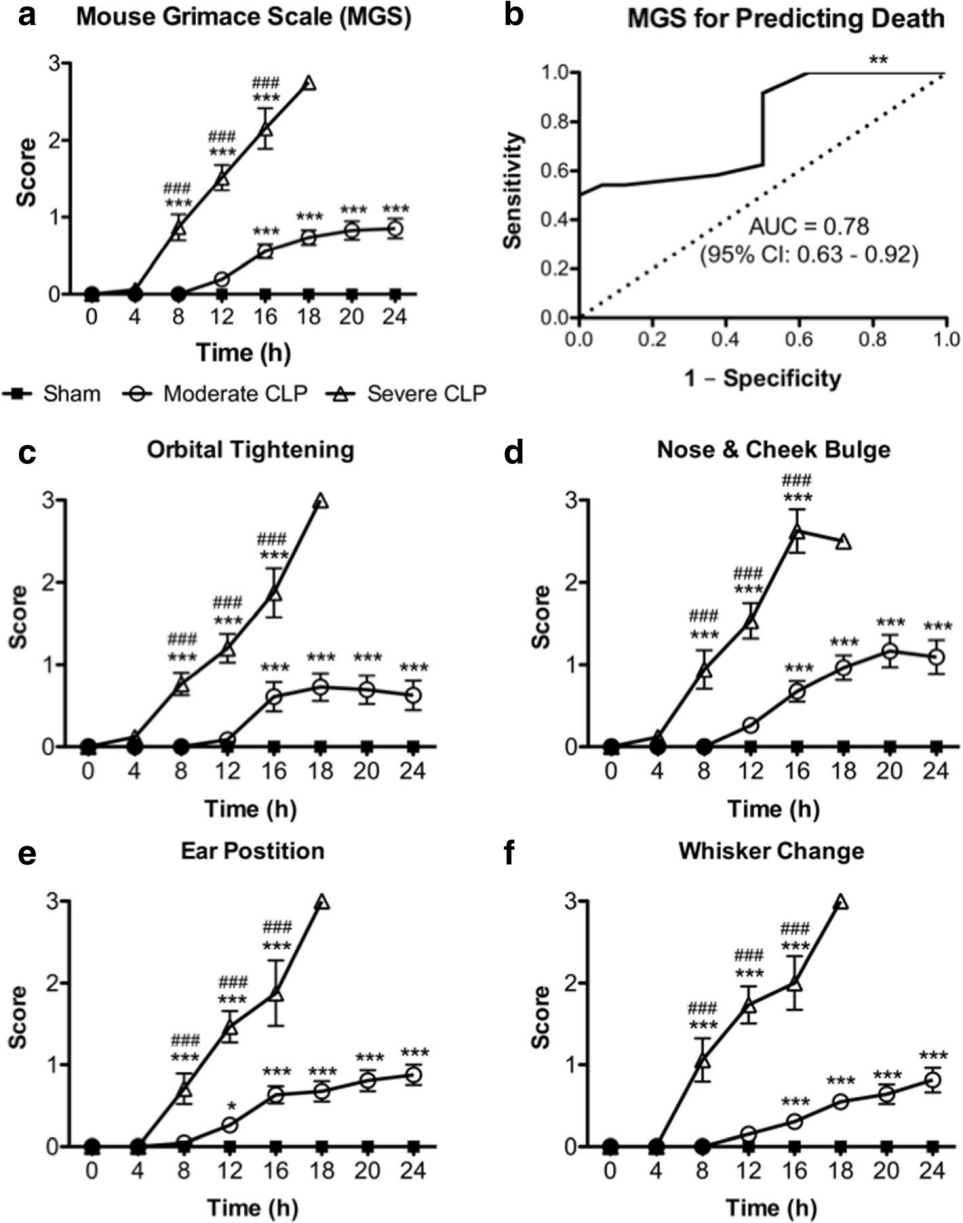
Modified Mouse Grimace Scale (MGS) score over time (**a**), MGS score as a predictor of death in septic mice (**b**), and MGS individual component scores (**c**–**f**) over time in mice undergoing moderate or severe CLP. Data are presented as mean ± SEM from six independent experiments representing a total number of *n* = 18 sham, *n* = 23 moderate CLP, and *n* = 17 severe CLP mice; *p < 0.05, **p < 0.01 (vs. line of identity in (**b**)), ***p < 0.001 vs. sham; ^###^*p* < 0.001 vs. moderate CLP, by two-way ANOVA. Note: Only two mice undergoing severe CLP survived past 18 h, therefore statistical comparisons were not performed at this time-point (**a**, **c**–**f**). Dotted line in (**b**) represents the line of identity for statistical comparison in ROC analyses. *AUC* area under the curve, *95*% *CI* 95% confidence interval

To assess the utility of the modified MGS for monitoring disease progression, we computed mean modified MGS scores as well as individual MGS component scores over time for each mouse undergoing moderate or severe CLP, or sham surgery, and further examined the ability of modified MGS scores to predict death using ROC analyses. Modified MGS scores were significantly higher as early as 8 h post-CLP in mice undergoing severe CLP sepsis compared to moderate CLP, whereas the latter group had significantly greater scores than sham mice starting 16 h post-surgery (Fig. 2a), which together indicates that modified MGS scores are useful for assessing disease progression over time in the CLP model of sepsis. However, modified MGS scores were not as robust (AUC = 0.78, 95% confidence interval 0.63–0.92) as temperature for predicting endpoint in the CLP model of sepsis (Fig. 2b). Each component score of the modified MGS—orbital tightening, nose and cheek bulge, ear position, and whisker change—demonstrated the same trends and differences between moderate and severe CLP groups as the overall modified MGS score (Fig. 2c–f), suggesting that each of these scores are equally useful for measuring disease progression. Collectively, these observations demonstrate that the modified MGS and its component scores may be useful for monitoring disease progression during CLP, but modified MGS is not sensitive and specific enough to predict death in murine CLP sepsis.

### Modified Murine Sepsis Score can assess disease progression and predict death in CLP sepsis

**Fig. 3:**
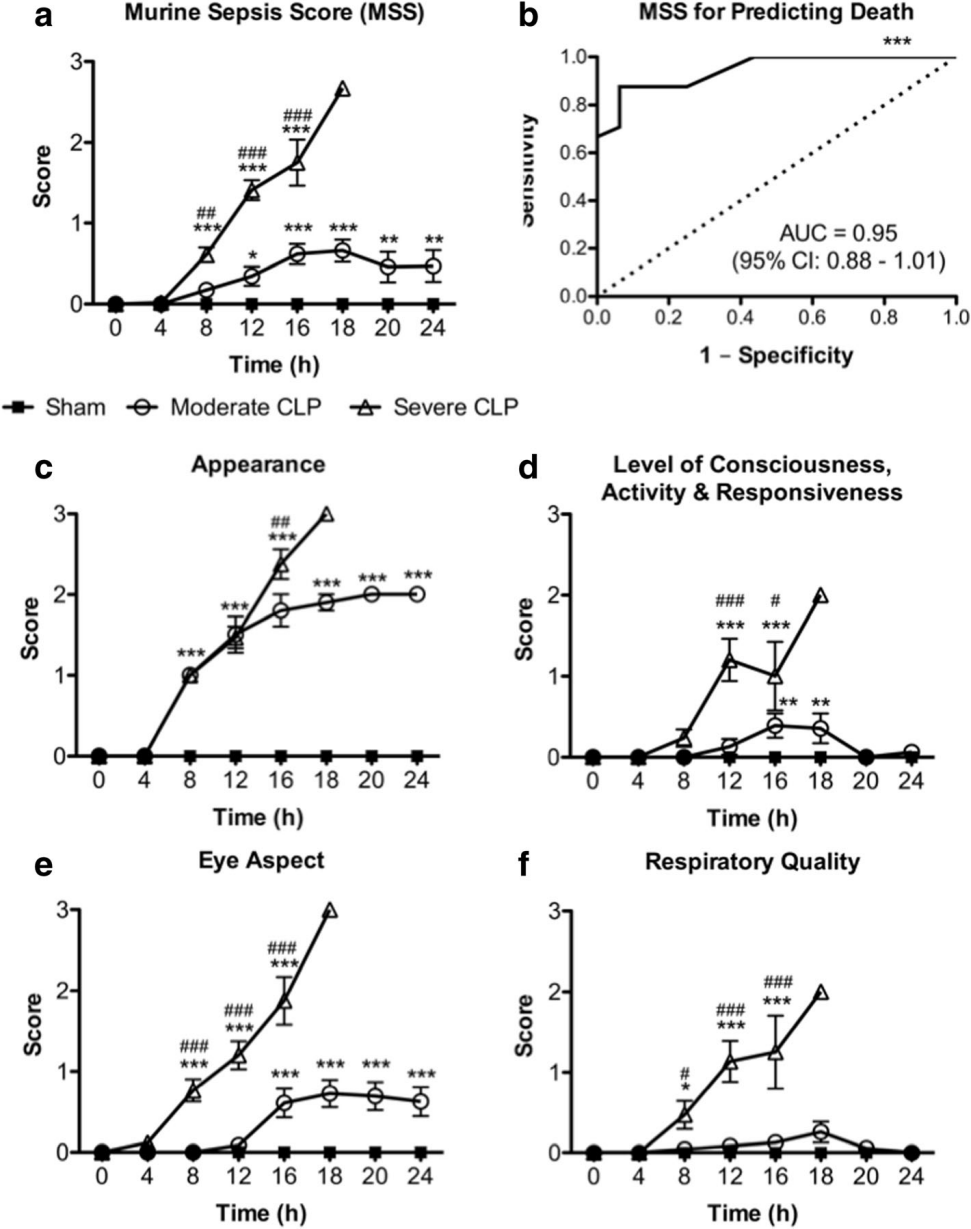
Modified Murine Sepsis Score (MSS) over time (**a**), MSS score as a predictor of death (**b**), and MSS individual component scores (**c**–**f**) over time in mice subjected to moderate or severe CLP. Data are presented as mean ± SEM from six independent experiments representing a total number of *n* = 18 sham, *n* = 23 moderate CLP, and *n* = 17 severe CLP mice; *p < 0.05, **p < 0.01, ***p < 0.001 vs. sham (or vs. line of identity in (**b**)); ^#^*p* < 0.05, ^##^*p* < 0.01, ^###^*p* < 0.001 vs. moderate CLP, by two-way ANOVA. Note: Only two mice undergoing severe CLP survived past 18 h, therefore statistical comparisons were not performed at this time-point (**a**, **c**–**f**). Dotted line in (**b**) represents the line of identity for statistical comparison in ROC analyses. Level of consciousness, activity, and response to stimuli scores were the same for each mouse, and therefore are displayed together in (**d**). *AUC* area under the curve, *95*% *CI* 95% confidence interval

To determine whether the modified MSS is useful for monitoring progression of disease and predicting death in CLP sepsis, we evaluated the overall modified MSS along with its individual component scores over time and computed an ROC curve to measure the predictive ability of the overall score. Similarly to the modified MGS scores, modified MSS was useful in distinguishing the progression of disease between severe and moderate CLP sepsis as early as 8 h post-CLP; however, the differences in modified MSS between moderate CLP and sham mice was significantly different from 12 h onward (Fig. 3a; vs. 16 h for modified MGS, Fig. 2a), suggesting that the modified MSS is more sensitive than modified MGS for assessing disease progression in CLP sepsis. Furthermore, the modified MSS was more robust (AUC = 0.95; 95% confidence interval 0.88–1.01) than temperature (Fig. 1d) for predicting death in the CLP model of sepsis utilized in this study (Fig. 3b). Interestingly, the individual score components of modified MSS appeared to have variable ability to differentiate between sham, moderate, and severe CLP sepsis groups over time. For instance, while “Appearance” was able to differentiate between sham and both CLP groups as early as 8 h post-surgery, it was not able to distinguish moderate CLP from severe CLP until 16 h (Fig. 3c)—at which time only 8 of 17 severe CLP mice remained alive (Fig. 1a). On the other hand, “Level of Consciousness, Activity, and Responsiveness” and “Respiratory Quality” were not able to consistently differentiate between sham and moderate CLP mice, but were useful for monitoring disease progression in severe CLP mice starting at 12 and 8 h post-CLP, respectively (Fig. 3d, f). The most useful component of the modified MSS for assessing disease progression in CLP sepsis appears to be the “Eye Aspect” score, which varied significantly between moderate and severe CLP groups from 8 h post-surgery onward, and between moderate CLP and sham groups from 16 h onward (Fig. 3e). Taken together, these data demonstrate that the modified MSS is effective for monitoring disease progression and for predicting death in our CLP model of sepsis.

### Modified Mouse Clinical Assessment Score for Sepsis can predict death in CLP sepsis

**Fig. 4:**
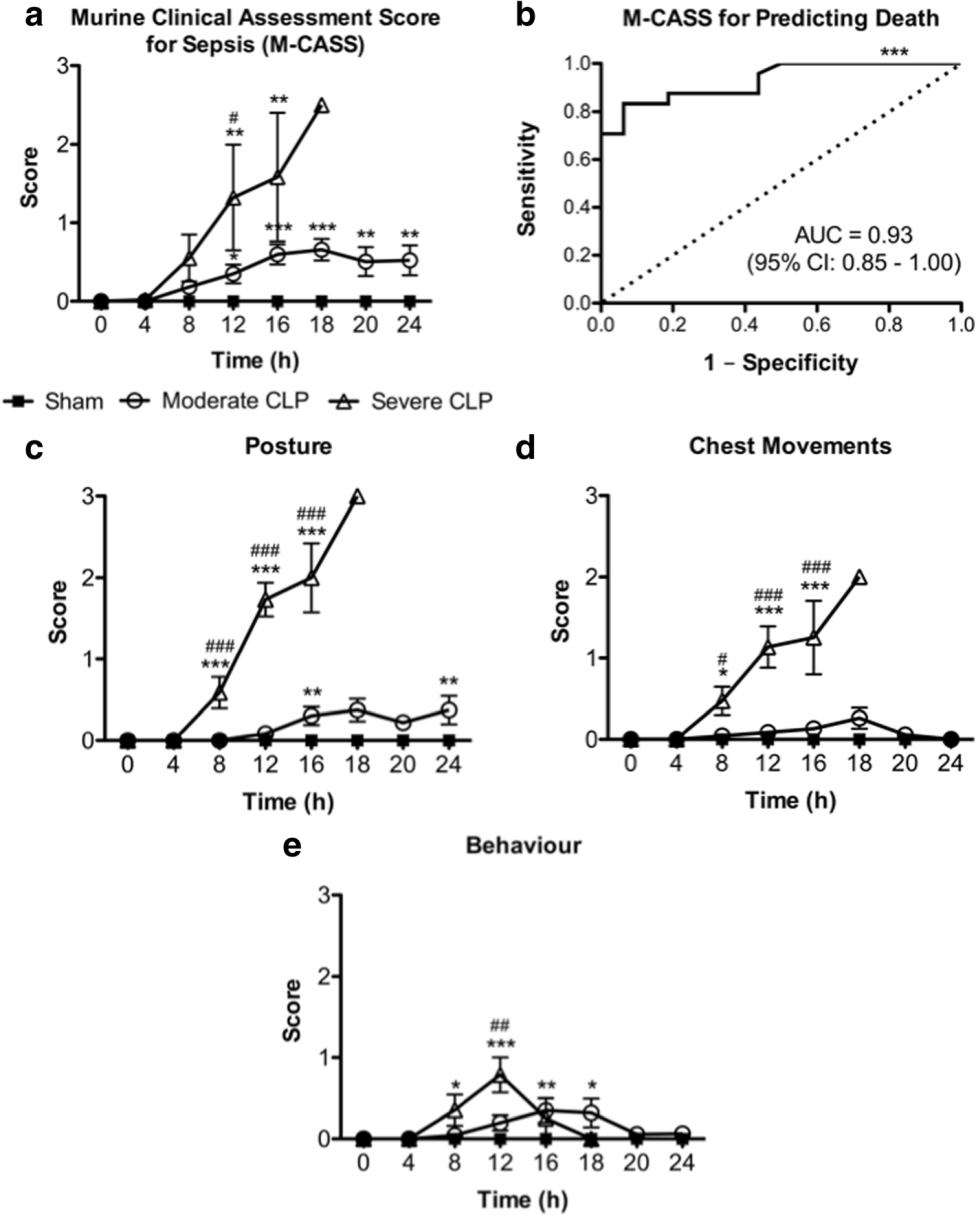
Modified Mouse Clinical Assessment Score for Sepsis (M-CASS) over time (**a**), M-CASS score as a predictor of death in septic mice (**b**), and individual M-CASS component scores (**c**–**f**) over time in mice undergoing moderate or severe CLP. Data are presented as mean ± SEM from six independent experiments representing a total number of *n* = 18 sham, *n* = 23 moderate CLP, and *n* = 17 severe CLP mice; *p < 0.05, **p < 0.01, ***p < 0.001 vs. sham (or vs. line of identity in (**b**)); ^###^*p* < 0.001 vs. moderate CLP, by two-way ANOVA. Note: Only two mice undergoing severe CLP survived past 18 h, therefore statistical comparisons were not performed at this time-point (**a**, **c**–**e**). Dotted line in (**b**) represents the line of identity for statistical comparison in ROC analyses. Fur Aspect, Activity, and Eyelid component scores are identical to scores in Fig. 3c–e, respectively, thus are not shown again here, but are included as part of the overall M-CASS score. *AUC* area under the curve, *95*% *CI* 95% confidence interval

In order to determine if the modified M-CASS system is useful for monitoring sepsis progression, we evaluated each component score as well as modified M-CASS scores over time in mice following sham surgery, moderate CLP, or severe CLP, and further assessed the ability of modified M-CASS scores to predict endpoint by conducting ROC analysis. As shown in Fig. 4a, modified M-CASS scores were able to differentiate disease progression between sham, moderate CLP, and severe CLP groups at 12 h post-surgery, albeit with notable variability in the scores of severe CLP mice. Overall, modified M-CASS scores had similar sensitivity and specificity (AUC = 0.93, 95% confidence interval 0.85–1.00) as modified MSS scores (Fig. 3b) for predicting death in mice subjected to moderate or severe CLP sepsis (Fig. 4b). The modified M-CASS and MSS systems share three similar component scores: (1) “Fur Aspect” and “Appearance” (respectively), (2) “Activity,” and (3) “Eye Aspect” and “Eyelids” (respectively), which resulted in identical scores for these components in both systems based on our standardization of each scoring system to a four-point grading scale (data shown in Fig. 3c–e). The remaining M-CASS component scores of “Posture,” “Chest Movements,” and “Behavior” demonstrated relatively poor ability to differentiate between sham and moderate CLP groups, and only “Posture” and “Chest Movements” were useful in discriminating between moderate and severe CLP sepsis (Fig. 4c–e). Therefore, while modified M-CASS appears to be useful for predicting death in CLP sepsis, these scores have greater variability than modified MSS for monitoring disease progression in our models of moderate and severe CLP sepsis.

## Discussion

In this study, we compared modified versions of the MSS [4], M-CASS [5], and MGS [6, 15] scoring systems that were standardized to a four-point grading scale, in order to determine which system would best serve as a surrogate endpoint for mortality in the gold standard CLP sepsis model. Our findings demonstrate that the modified MSS—a composite score monitoring Appearance, Level of Consciousness, Activity, Response to Stimuli, Eye Aspect, and Respiratory Quality [4]—is the most useful of these scoring systems for distinguishing between moderate and severe CLP models, and for predicting death in mouse CLP sepsis. In contrast, modified MGS was effective for tracking the severity of disease in our CLP model, but was not as robust as modified MSS for predicting death. On the other hand, modified M-CASS was effective at predicting death in our CLP sepsis model, but was more variable than the other two scores for differentiating disease severity in septic mice. Due to the semi-quantitative and subjective nature of all three scoring systems, we monitored body temperature as an objective vital sign: our data show that temperature decreases over time in a sepsis severity-dependent manner and that reduced body temperature is a robust predictor of death in the CLP mouse model of sepsis—a novel and important finding. Recent studies by Lewis et al. have shown that body temperature tracking with an implanted wireless biotelemetry device is useful for monitoring the physiologic response to antibiotic treatment and fluid resuscitation in murine sepsis [25], which is consistent with our findings that body temperature can be used to monitor disease severity in CLP sepsis.

Based on our observations, we recommend that these surrogate markers be used in murine sepsis studies for several reasons. First, these tools offer a general non-invasive assessment of disease progression and are amenable to high monitoring frequency, unlike repetitive blood sampling which has been shown to reduce red blood cell count and hemoglobin concentrations after 5 days of sampling only 35 μL daily [26]. Second, evaluation of symptoms provides easily accessible information regarding the change in an animal’s health status, facilitating macroscopic monitoring of the physiologic response when testing novel therapeutic interventions or effects of comorbidities. Lastly, monitoring of vital signs improves the clinical relevance of experimental sepsis studies, and our study shows that core body temperature measured using a rectal thermometer is an objective marker of sepsis progression and death. Therefore, we recommend that body temperature be routinely monitored in CLP studies and, based on our data, body temperature below 30 °C or a reduction in body temperature of > 5 °C could be utilized as thresholds to replace death as an endpoint in mouse CLP studies involving male C57Bl/6J mice.

To maximize the relevance of this study, we took several factors into consideration when selecting which variation of the CLP model to utilize. For instance, the severe model of CLP implemented in this study resulted in 100% mortality (i.e., sacrifice at endpoint) within 24 h, a finding consistent with mortality rates reported during initial development of the CLP procedure [19]. Furthermore, our moderate severity CLP model resulted in 30% mortality, which is consistent with typical mortality rates observed in septic patients with multiple organ dysfunction [27, 28]. In both CLP models, early and aggressive fluid resuscitation was given to prevent shock and rapid death due to circulatory collapse [29]. Additionally, buprenorphine analgesic at the upper range of the recommended dose was given every 4 h, in order to minimize pain and the potential confounder of facial grimacing as a pain response rather than a reflection of disease progression. Previous studies have demonstrated that buprenorphine analgesia alone has minimal effects on the MGS scores in mice [30], which is consistent with our observations of normal MGS scores in sham mice throughout the study period, and taken together with our sham MSS and M-CASS data, suggests that these observations likely extend to the MSS and M-CASS scoring systems also.

There are some limitations to our CLP model, which should be considered when interpreting the results of our study in the context of other variations to the CLP model. For instance, antibiotics were not administered in order to allow for the natural course of sepsis pathophysiology to progress over a manageable study period of 24 h, since mice had to be monitored every 4 h (including overnight) over the entire study duration in accordance with ethics approval by our Animal Research Ethics Board and CCAC standards. Furthermore, in order to induce varying degrees of sepsis severity in our CLP model in a 24-h study period, we extruded varying amounts of fecal matter into the peritoneal cavity in our 18-gauge double-puncture model, rather than other commonly utilized variables such as cecal ligation length [17]. The shorter study duration may have also impacted the utility of the modified M-CASS for monitoring disease progression, as we excluded “Weight Loss” and “Chest Sounds” components from the modified composite M-CASS score due to undetectable changes in these components over the 24-h study period. In other CLP models where sepsis develops over 3–10 days [17], weight loss and chest sounds may be effective components of the M-CASS scoring system. Therefore, the utility of M-CASS and MSS should be validated over longer study periods that employ more mild CLP models, and with administration of antibiotics, in order to determine the widespread applicability of these scoring systems as surrogate markers of death in CLP studies. Finally, similar studies should be conducted in rat models of CLP sepsis to determine which surrogate markers are appropriate to replace death in other widely applicable animal sepsis models.

## Conclusions

In summary, the modified MSS and body temperature measurements are effective, clinically relevant methods for monitoring endpoint, assessing sepsis progression, and predicting death in our mouse model of acute CLP sepsis. Further efforts to use techniques such as these scoring systems, rather than death as an endpoint, should be made to meet the increasing standards for conducting ethical and humane research using murine models to study sepsis. Finally, based on our observations, body temperature monitoring should be considered to replace death as an endpoint in future mouse CLP studies.

## Abbreviations

ANOVA: Analysis of variance
ARRIVE: Animal Research: Reporting of In Vivo Experiments
AUC: Area under the curve
CCAC: Canadian Council on Animal Care
CLP: Cecal ligation and puncture
M-CASS: Mouse clinical assessment score for sepsis
MGS: Mouse grimace scale
MSS: Murine sepsis score
ROC: Receiver operating characteristic
SEM: Standard error of the mean
